# Supplementary material for: Determination of the Optimal Landmark for Tube Thoracostomy in Trauma Patients: A Retrospective Study
Source: J Clin Med. 2025 Oct 25;14(21):7571. doi: 10.3390/jcm14217571 (PMC12610840; doi:10.3390/jcm14217571)
Supplement: Supplementary file 1 [file jcm-14-07571-s001.zip › jcm-3881499-supplementary.pdf]

Supplementary Material – Detailed Methodology for Measurement

Title: Determination of the Optimal Landmark for Tube Thoracostomy in Trauma Patients: A Retrospective Study

Journal Name: *Journal of Clinical Medicine*

Author Names

Mina Lee [1], Jaeik Jang [2,*], Jae-Hyug Woo [1,3,*], Hyuk Jun Yang [1,3], Woo Sung Choi [1,3], Jae Ho Jang [1,3] and Sung Youl Hyun [2,3]

Affiliation

1 Department of Emergency and Critical Care Medicine, Gachon University Gil Medical Center, Incheon 21565, Republic of Korea

2 Department of Traumatology, Gachon University Gil Medical Center, Incheon 21565, Republic of Korea

3 Gachon University College of Medicine, Incheon 21565, Republic of Korea

* Correspondence: blanc7654@gmail.com (J.J.); emmetalkiller@gilhospital.com (J.-H.W.); Tel.: +82-32-460-3901 (J.-H.W.)

p.3 **S1. Computed Tomography Acquisition Protocol**

p.3 **S2. Measurement Procedures**

p.3 **S2.1. Mid-Axillary Line**

p.4 **S2.2. Mid-Sternum Method**

p.9 **S2.3. Mid-Arm Point Method**

p.10 **S2.4. Nipple Method**

p.11 **S2.5. Fifth ICS Method**

S1. Computed Tomography Acquisition Protocol

- **Computed tomography (CT) scanner**: 128-slice CT scanner (Siemens Healthineers)
- **Slice thickness**: 3.00 mm
- **CT protocol**: Chest CT for trauma
- **Patient positioning**: Supine position with arms down
- **Reconstructed planes**: Axial, sagittal, and coronal planes

S2. Measurement Procedures

All measurements were performed using the hospital's Picture Archiving and Communication System (PACS) with the two-dimensional (2D) line and three-dimensional (3D) cursor tools. The procedures were as follows:

S2.1 Mid-Axillary Line

The reference point was set as the most laterally located pleural point (Figure S1A; cross), and this was identified on the axial plane at the level of the xiphoid process (Figure S1A; arrowhead). A vertical cranio-caudal scout line drawn from this point was defined as the mid-axillary line (MAL) (Figure S1A, yellow line: automatically generated scout lines from the cross (+) point; Figure S1B, red line: xiphoid process level; Figure S1B, yellow line: MAL).
This defined MAL was used as the reference line for determining the possible insertion sites at the mid-sternum, mid-arm, nipple, and 5th intercostal space (ICS) levels.

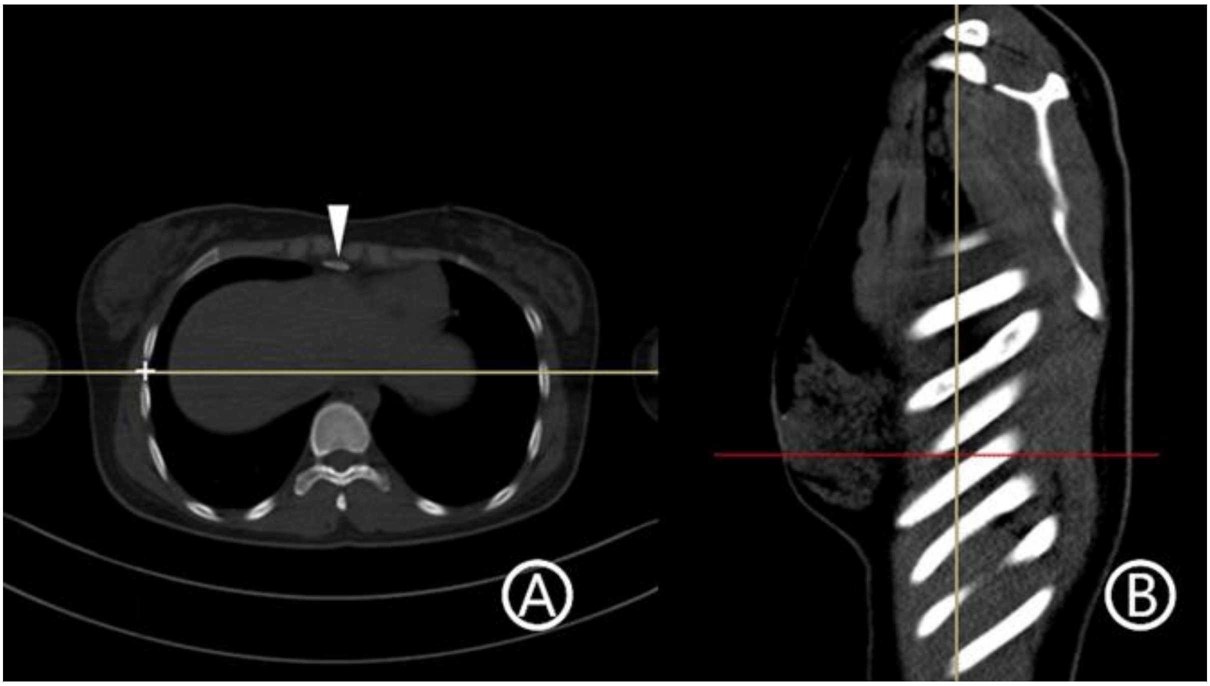

Supplementary Figure S1. Definition of the mid-axillary line (MAL) on computed tomography. (**A**) Axial image showing the most lateral pleural point (cross) at the xiphoid level (arrowhead), from which a horizontal scout line (yellow line) was generated. (**B**) Sagittal image showing the xiphoid level (red line) and the corresponding MAL (yellow line).

S2.2 Mid-Sternum Method

1. **Mid-sternum point**: On the mid-sagittal plane (Figure S2A), the lengths of the manubrium (from the jugular notch to the sternal angle; Figure S2A, L1) and the body–xiphoid segment (from the sternal angle to the xiphoid process; Figure S2A, L2) were measured using the 2D line tool. The total length was defined as the sum of the length of the manubrium and that of the body–xiphoid segment. The midpoint of the total length was defined as the mid-sternum point (Figure S2A; arrowhead). A horizontal line through the mid-sternum point on the MAL-marked sagittal plane, shown as the red line in Figure S2A, was intersected with the MAL, shown as the yellow line in Figure S2A. This location was designated with a 3D cursor, shown as the solid dot in Figure S2A. The possible insertion site and route at the mid-sternum level were identified using the cross-link function in the coronal (Figure S2B) and axial planes (Figure S2C).

2. **Possible insertion site**: The trajectory of the MAL level (Figure S2C; yellow line) was identified on the axial plane at the mid-sternum level. The pleural point located at the most peripheral end of this trajectory was defined as the possible insertion site (Figure S2C; cross).

3. **Possible insertion route**: A horizontal scout line passing through the possible insertion site (Figure S2B, red line; Figure S2C, yellow line).

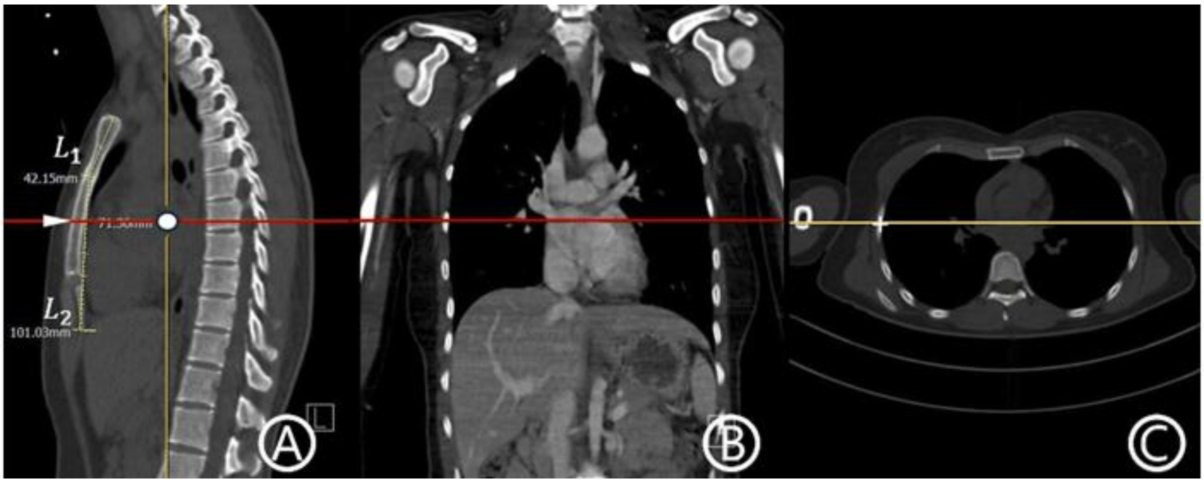

Supplementary Figure S2. Determination of the mid-sternum level and identification of the possible insertion site and route. (A) The mid-sternum point (arrowhead) was defined as the midpoint of the sum of the lengths of the manubrium (L1) and the body–xiphoid segment (L2) on the mid-sagittal plane; a horizontal red line through this point intersected the yellow mid-axillary line (MAL)(solid dot). (B) Coronal image showing the possible insertion route (red line) passing through the mid-sternum level. (C) Axial image at the mid-sternum level showing the trajectory of the MAL level (yellow line); the pleural point located at the most peripheral end of this trajectory was designated as the possible insertion site (cross).

4. **Intercostal level of the possible insertion site**: Using the 3D cursor and cross-link function, the ICS or rib level of the insertion site was identified on the sagittal plane (Figure S3; cross).

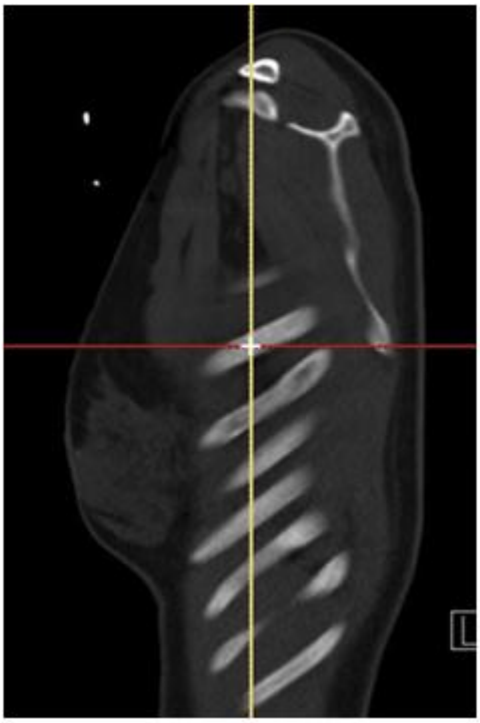

Supplementary Figure S3. Intercostal level of the possible insertion site. Red line: mid-sternum level; yellow line: mid-axillary line; cross: possible insertion route.

5. **Chest wall thickness and presence of breast tissue**: The presence of breast tissue along the possible insertion route was assessed along the axial plane at the mid-sternum level, and the distance from the skin to the pleura was measured (Figure S4, yellow line: possible insertion route; Figure S4, L: chest wall thickness).

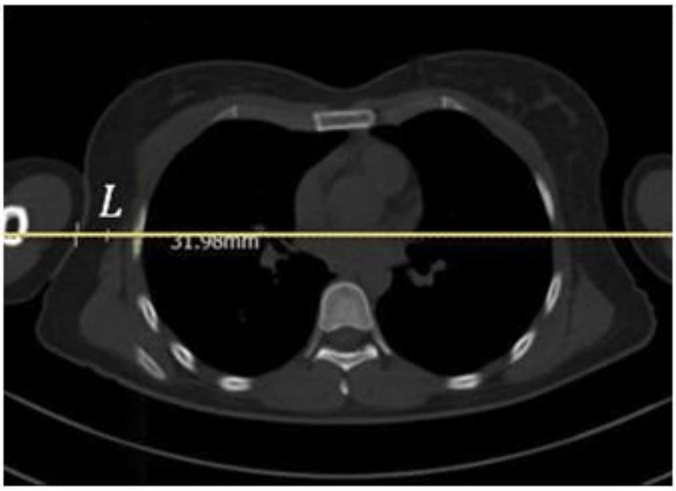

Supplementary Figure S4. Measurement of chest wall thickness and presence of breast tissue. Yellow line: possible insertion route; L: chest wall thickness

6. **Distance to the pectoralis major**: The perpendicular distance from the insertion route indicated by the solid dot in Figure S5 to the pectoralis major muscle indicated by the arrowhead in Figure S5 was measured (Figure S5; L).

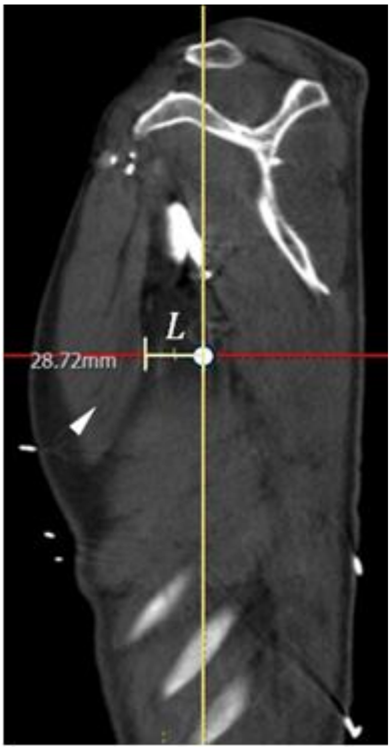

Supplementary Figure S5. Measurement of the distance to the pectoralis major. Red line: mid-sternum level; yellow line: mid-axillary line, Arrowhead: Pectoralis major; L: distance from the possible insertion route to Pectoralis major; solid dot: possible insertion route.

7. **Distance to the latissimus dorsi**: The perpendicular distance from the insertion route indicated by the solid dot in Figure S6 to the latissimus dorsi muscle indicated by the arrowhead in Figure S6 was measured.

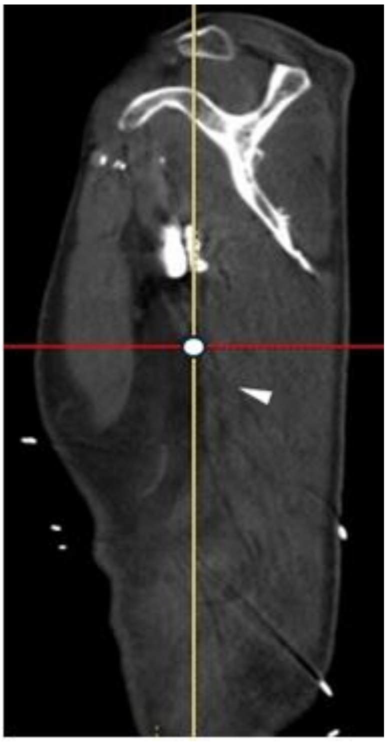

Supplementary Figure S6. Measurement of the distance to the latissimus dorsi. Red line: mid-sternum level; yellow line: mid-axillary line; Arrowhead: Latissimus dorsi; solid dot: possible insertion route.

8. **Distance to the highest diaphragm at the MAL level**: On the coronal image at the MAL level, the vertical distance between the possible insertion route and the highest point of the diaphragm was measured (Figure S7; L).

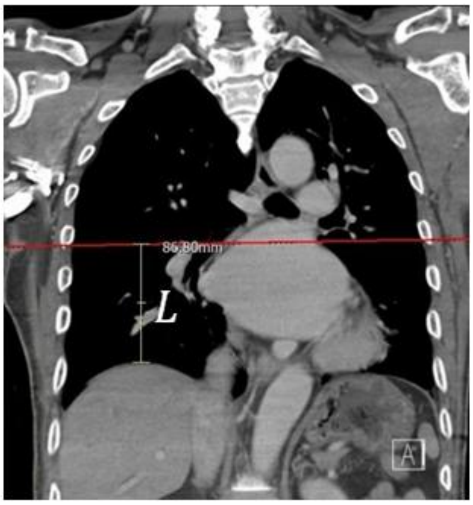

Supplementary Figure S7. Measurement of the distance to the highest diaphragm point at the mid-axillary line level (L). Red line: possible insertion route.

9. **Cranio-caudal distance to the highest diaphragm in any coronal plane**: Among all coronal images, the image showing the highest diaphragm elevation was selected. The cranio-caudal distance between this highest point and the possible insertion route was measured as indicated by L in Figure S8 to assess whether the route penetrates the diaphragm or enters abdominal organs.

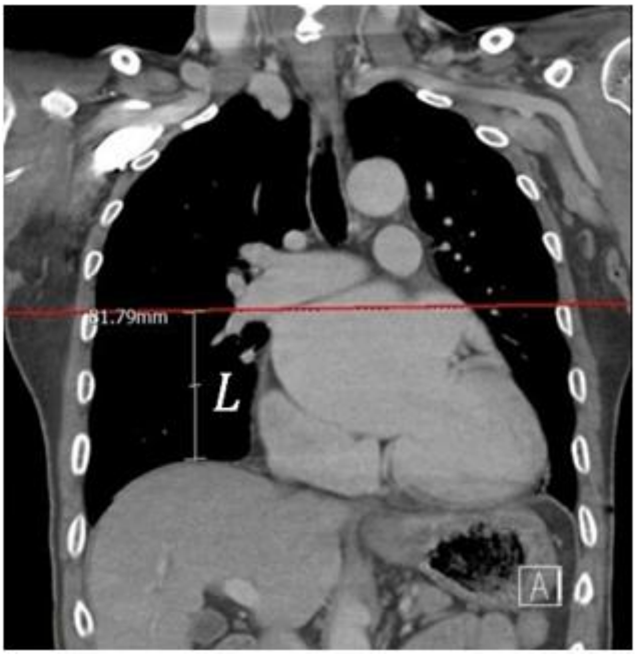

Supplementary Figure S8. Cranio-caudal distance to the highest diaphragm point across coronal planes (L). Red line: possible insertion route

10. **Additional assessments**: All measurements were performed bilaterally, and we recorded the number of ribs involved, the presence of subcutaneous emphysema, traumatic pneumothorax, traumatic hemothorax, sternal fracture, rib fracture, fractured rib levels, and multiple rib fractures

S2.3 Mid-Arm Point Method

1. **MAL definition**: As described in Section 2.1 (Figure S1).
2. **Mid-arm point**: The distance between the acromion process (Figure S9A; P1) and the olecranon (Figure S9A; P2) was measured using the 2D line tool on the chest CT topogram. The midpoint of the measured linear distance was defined as the mid-arm point (Figure S9A; arrowhead). A horizontal line, shown as the red line in Figure S9B, was drawn through the mid-arm point on the sagittal plane with the MAL (Figure S9B; yellow line). The line's intersection with the MAL is represented by the solid dot in Figure S9B was designated with a 3D cursor. The possible insertion site and route were identified using the cross-link function on the coronal and axial planes (Figure S9C, red line; Figure S9D, yellow line).

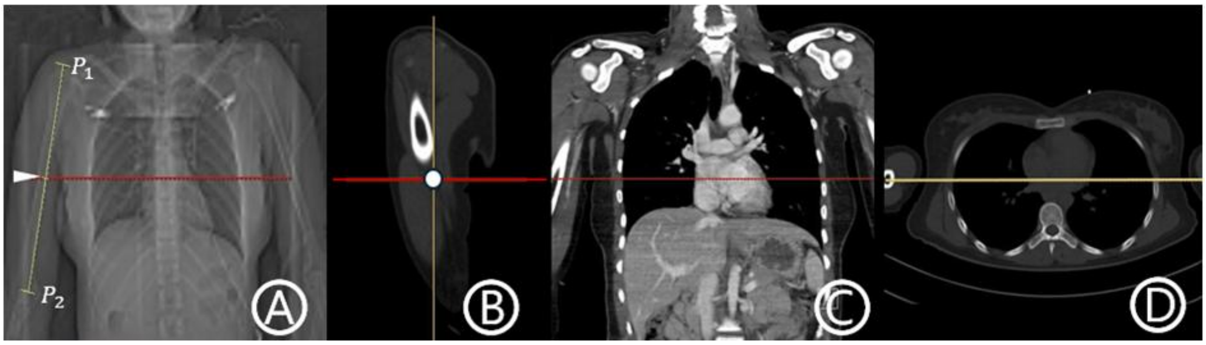

Supplementary Figure S9. Determination of the mid-arm point and identification of the possible insertion site and route. (**A**) The distance between the acromion (P1) and olecranon (P2) was measured on the chest CT topogram, and the midpoint of the measured distance was defined as the mid-arm point (arrowhead); the red line indicates the mid-arm level. (B) Sagittal image showing a horizontal red line through the mid-arm point intersecting the mid-axillary line (MAL, yellow line); the intersection was marked with a 3D cursor (solid dot). (C) Coronal and (D) axial images showing the projected possible insertion route based on the cross-link function (red line in C, yellow line in D). CT, computed tomography; 3D, three-dimensional.

3. **Subsequent measurements**: All variables related to the possible insertion site and route were assessed in the same manner as in the mid-sternum method.

S2.4 Nipple Method

1. **MAL definition**: As described in Section 2.1 (Figure S1).

2. **Nipple level point**: On the sagittal plane with the MAL indicated (Figure S10A; yellow line), a horizontal line passing through the nipple marked by the arrowhead in Figure S10A was drawn (Figure S10A; red line), and the intersection with the MAL represented by the solid dot in Figure S10A was designated using the 3D cursor. The possible insertion site and route were identified using the cross-link function on the coronal and axial planes (Figure S10B, red line; Figure S10C, yellow line).

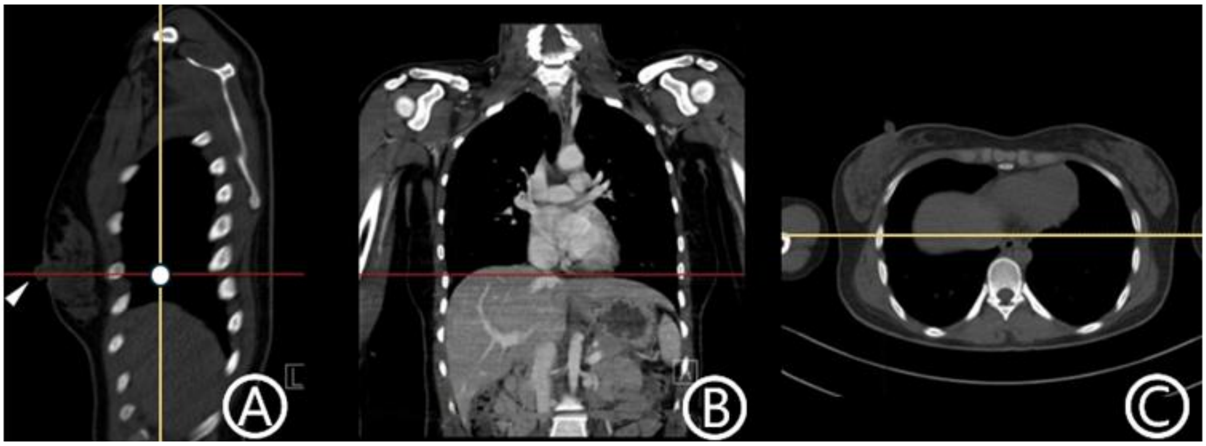

Supplementary Figure S10. Determination of the nipple level point and identification of the possible insertion site and route. (**A**) A horizontal red line was drawn through the nipple level (arrowhead) on the sagittal plane, and its intersection with the mid-axillary line (represented by the yellow line) was marked using a 3D cursor (solid dot). (**B**, **C**) The possible insertion site and route were identified using the cross-link function on the coronal (B, red line) and axial (C, yellow line) planes. 3D, three-dimensional.

3. **Subsequent measurements**: All variables related to the possible insertion site and route were assessed in the same manner as in the mid-sternum method.

S2.5 Fifth ICS Method

1. **MAL definition**: As described in Section 2.1 (Figure S1).

2. **Fifth ICS identification**: On the sagittal plane, the space between the fifth rib and the sixth rib was defined as the fifth ICS (Figure S11A, P1: fifth rib; Figure S11A, P2: sixth rib). A horizontal line passing through this space, represented by the red line in Figure S11A, was intersected by the MAL (Figure S11A; yellow line). The line's intersection point was marked with a 3D cursor (Figure S11A; solid dot). The possible insertion site and route were identified using the cross-link function on the coronal and axial planes (Figure S11B, red line; Figure S11C, yellow line).

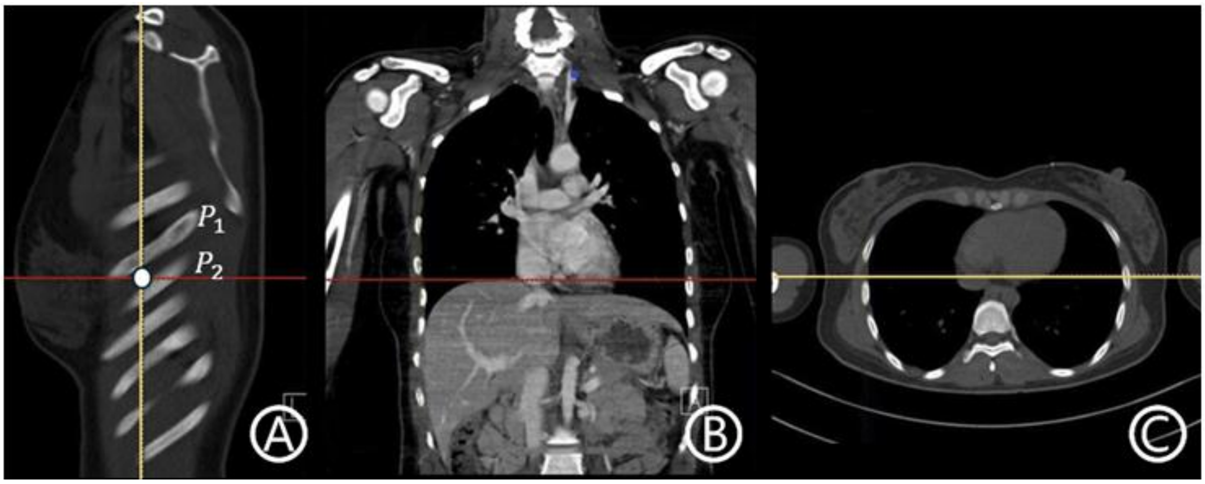

Supplementary Figure S11. Identification of the fifth intercostal space and the possible insertion site and route. (**A**) On the sagittal plane, the fifth intercostal space was defined as the space between the fifth rib (P1) and sixth rib (P2); a horizontal red line passing through this space intersected the mid-axillary line (yellow line), and the intersection point was marked using a 3D cursor (solid dot). (**B**, **C**) The possible insertion site and route were identified using the cross-link function on the coronal (**B**, red line) and axial (**C**, yellow line) planes. 3D, three-dimensional.

3. **Subsequent measurements**: All variables related to the possible insertion site and route were assessed in the same manner as in the mid-sternum method.
